# The Effect of Normobaric Hypoxia in Middle- and/or Long-Distance Runners: Systematic Review

**DOI:** 10.3390/biology11050689

**Published:** 2022-04-30

**Authors:** Inés Albertus-Cámara, Vicente Ferrer-López, Ignacio Martínez-González-Moro

**Affiliations:** Research Group of Physical Exercise and Human Performance, Mare Nostrum Campus, University of Murcia, 30100 Murcia, Spain; ines.albertusc@um.es (I.A.-C.); vferrer@um.es (V.F.-L.)

**Keywords:** endurance runners, normobaric hypoxia, sports performance, altitude

## Abstract

**Simple Summary:**

Exposure to normobaric hypoxia, that is, breathing air with a decreased proportion of oxygen without changing the atmospheric pressure, is a resource used in endurance runners with the aim of increasing their athletic performance. This systematic review describes the effects of different hypoxia programmes, exposure time and duration of the intervention on some haematological and sporting performance parameters. It has been shown that prolonged exposures to hypoxia are necessary to increase haemoglobin values. However, shorter sessions (less than three hours) are effective in increasing time to exhaustion. This review may be useful for planning the training of runners subjected to normobaric hypoxia sessions.

**Abstract:**

Background: The use of normobaric hypoxia can bring benefits to sports performance because it improves haematological parameters and/or physical activity tests. Our objective was to conduct a systematic review so as to analyse the methods used in hypoxia and to detect its effects on middle- and/or long-distance runners. Methods: Research was conducted using five electronic databases (PubMed, SportDiscus, Cochrane Library, Scopus and PEDro) until December 2021. The methodological quality of the included studies was assessed using the PEDro scale. Results: Having analysed 158 studies, 12 were chosen for the qualitative and quantitative synthesis. A significant improvement on time until exhaustion was detected, and oxygen saturation decreased after the intervention. There were no significant changes in the 3000-metre time trial or in the haematocrit percentage. The changes in percentage of reticulocytes, heart rate, maximal heart rate, lactate concentration and erythropoietin were heterogeneous between the different research studies. Conclusion: short exposure (less than 3 h to normobaric hypoxia significantly increases the time to exhaustion). However, longer exposure times are necessary to increase haemoglobin. Altitude and exposure time are highly heterogeneous in the included studies.

## 1. Introduction

With an increase in altitude, the barometric pressure decreases exponentially, which leads to a progressive reduction in the ambient partial pressure of oxygen (PO_2_), otherwise called hypobaric hypoxia (HH) [1]. This hypoxia can be injurious to health, depending on the altitude attained, rate of ascent, duration at altitude, and physical activity among other factors. It is known that at altitudes of 2.438 to 3.048 m, there is an acute hypoxic ventilatory response (HRV) causing hypocapnia and respiratory alkalosis, which endanger blood-oxygen supply and brain blood flow (2.438 to 4572 m) [2].

However, hypoxia also has the ability to foster a series of adaptations, such as: increased capability for transporting oxygen per unit of blood; increased oxygen supply for a given cardiac output [1]; improved glycolytic enzymes, glucose transport and pH regulation [3,4] or improved adaptations gained from resistance training [5,6].

In elite sport, the difference in performance between athletes is minimal [7]. To obtain a competitive advantage, many endurance athletes, such as distance runners and road cyclists, subject themselves to regular altitude and hypoxic training using the different strategies available [8,9,10].

The traditional method of doing so is to train on mountains, but not all the athletes or teams can access high-altitude areas to train. Therefore, there has recently been an increase in the number of techniques designed to “take the mountain to the athlete”. Nitrogen houses, hypoxia stores and special devices, such as facial masks, are some of the options that can simulate high-altitude conditions, and as a result, induce states of hypoxia [11]. This type of state is called normobaric hypoxia, and is characterised by a reduction in the proportion of air oxygen without changing atmospheric pressure (in hypobaric hypoxia it does decrease) [11]. Due to these practical and logistical factors, normobaric hypoxia (NH) is frequently used as a laboratory alternative to hypobaric hypoxia [1]. 

There are different methods of applying hypoxia that are related to training. “Living high–training high” (LHTH) and “living high–training low” (LHTL) are two techniques that require relatively long exposure times (12 h per day for a minimum of 2 weeks) so as to accumulate an hypoxic dose [12,13]. Athletes frequently subject themselves to sleep in hypoxia chambers [14] or undertake the preseason in high-altitude areas [15]. The hypoxic training method known as “living low–training high“(LLTH) is gaining popularity because athletes live in normoxia and carry out their training sessions in hypoxic conditions. This type of exposure has a duration of 3 h and takes place two to five times per week, and therefore does not provide a hypoxic stimulus to introduce hematologic changes, as is the case with the LHTH and LHTL methods [12].

As an alternative to existing methods, the intermittent normobaric hypoxia method, with a duration of less than 3 h, was developed. The objective of this method was to obtain the advantages of the models LHTH and LHTL with less exposure time and an intermittent mode [16]. This model is the subject of controversy, with some authors claiming that it improves exercise performance in subelite athletes, but not in elite athletes [17], while other authors [16] claim that it does not lead to any physiological adaptation. 

## 2. Objectives 

As mentioned above, the aim of this systematic review was to analyse the methods used in hypoxia and to detect its effects on middle- and/or long-distance runners. Specifically, we aimed to answer the following questions, all of which relate to the use of normobaric hypoxia in endurance runners:

Research Question 1. Does hypoxia training increase time to exhaustion in runners?

Research question 2. Does exposure to normobaric hypoxia improve haematological parameters in runners? 

Research Question 3. What altitudes and exposure times are being used to increase athletic performance in runners? 

## 3. Material and Methods

This review was realized in accordance with the Campbell Collaboration policies and guidelines for systematic reviews and The Preferred Reporting Items for Systematic Reviews and Meta-Analysis (PRISMA) guidelines [18]. 

The review was previously registered with PRÓSPERO 2020 CRD4202021109.

### 3.1. Elegibility Criteria

#### 3.1.1. Types of Studies 

Seven randomised controlled trials (RCTs) and five quasi-experimental studies were included (*n* = 12; participants: *n* = 202)

We restricted study eligibility by language. We did not restrict study eligibility by publication status. However, all studies comprising this systematic review have been published.

#### 3.1.2. Types of Participants

We included studies with middle- and/or long-distance runners. We included studies that recruited both men and women, and studies that recruited men and women separately.

#### 3.1.3. Language

Articles published in English or Spanish were included.

#### 3.1.4. Publication Date

The search covered publication dates from the beginning until December 2021.

#### 3.1.5. Exclusion Criteria

Studies that did not consider measures of sports performance or haematological parameters, and studies that included athletes with illness or injury were excluded.

#### 3.1.6. Types of Interventions

- Hypoxia at rest or during treadmill exercise
- Hypoxia at rest or during cycloergometer exercise
- Intermittent normobaric hypoxia or long exposure to normobaric hypoxia

#### 3.1.7. Outcome Measure

• Primary
○ Time until exhaustion
• Secondary
○ Haematological parameters
○ Altitude and time under hypoxia

### 3.2. Data Sources and Search Strategy 

We searched in PubMed, SportDiscus, Cochrane Library, Scopus and PEDro database for relevant articles. 

Thus, we used only reliable, peer-reviewed databases, platforms and sources with search tools that allowed us to access the study dates, and thereby systematically identify studies. We checked the reference lists of all the included studies and systematic reviews for additional references.

The terms used to search the database were: (normobaric hypoxia OR altitude) AND runners.

### 3.3. Selection of Studies

Two review authors independently screened the titles and abstracts of all the retrieved references in Excel (Microsoft Excel 2018 for Windows). The full-text study reports were retrieved for all the citations that at least one review author considered potentially relevant. Two review authors independently screened the full-text articles and identified studies for inclusion; they also identified and recorded the reasons for excluding studies in the excluded studies characteristics. Any disagreements were resolved through discussion. The selection process is detailed in a PRISMA flow diagram [18].

### 3.4. Data Extraction and Management

We used a standardized piloted data collection form in Microsoft Excel 2018 for Windows and extracted the following study characteristics and outcome data: (i) Methods: study design; (ii) Participants: randomized number, study participants’ mean age or age range, study location and setting, recruitment methods, inclusion and exclusion criteria, and type of endurance sport; (iii) Interventions: a description of the training intervention characteristics, the dose and duration of the training intervention; (iv) Outcomes: a description of the primary and secondary outcomes in the review that were reported in the trial, and a listing of other outcomes collected in the trial; (v) Notes: the trial funding and any notable conflicts of interest of the trial authors. Two review authors independently extracted the outcome data from the included studies into Microsoft Excel 2018 spreadsheets, and compared the data to identify any discrepancies in the data entries. Any disagreements were resolved by consensus.

### 3.5. Methodological Quality Assessment

The methodological quality of each of the studies was carried out with PEDro Scale (Table 1) [19]. The maximum score was 7/10 [20,21] and the minimum was 3/10 [22,23]. The remainder of the articles ranged from 4/10 to 6/10 in their score.

## 4. Results 

### 4.1. Description of the Studies 

#### 4.1.1. Search Results

**Figure 1:**
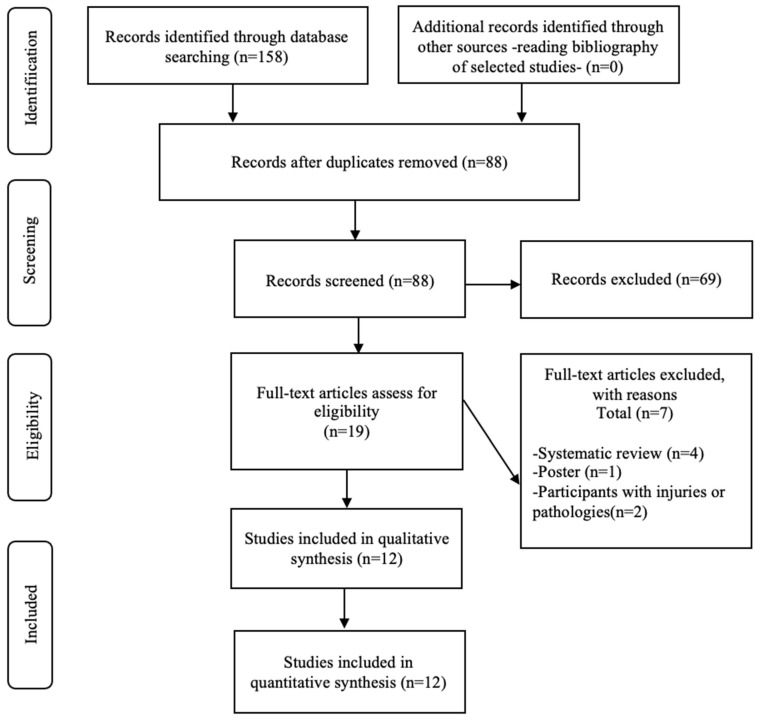
Preferred Reporting Items for Systematic Reviews and Meta-Analysis (PRISMA) diagram of the study screening process for examining the effect of intermittent normobaric hypoxia in runners.

The initial research comprised 158 studies. After removing duplications, 88 article titles and abstracts were reviewed. Following this process, 19 (full-text) were thoroughly read and, from these, 12 were finally included for the systematic review. The PRISMA flowchart illustrates the search and selection process (Figure 1).

#### 4.1.2. Included Studies 

Twelve studies made up this systematic review (Table 2). It is important to highlight that some of these presented several intervention groups, and that for each of these groups, the procedures involved differences; for instance, in the time of day at which the hypoxia was administrated (night/day), in the position (repose and/or physical activity), or in the time and manner of administration of hypoxia. 

Only eight of the scientific studies had a control group for their interventions [20,22,24,25,27,28,29,31]. The control groups of the studies [20,22,24,27,28] did not receive the hypoxia at rest, while their corresponding intervention groups did. Furthermore, the control groups of Neya et al. [29] and Dufour et al. [25] were physically active, but with an inspiratory pressure of 20.9%, both of which factors are considered normoxia conditions.

- Study location

The studies took place in different countries: four in Japan [26,27,28,29], two in Australia [30,31], two in France [22,25], one in The United States [20], one in Russia [23], one in Austria [24] and one in England [21].

- Sample size and years of studies

The sample size of each of the studies had a variation of 7 to 29 subjects. The oldest studies dated from 2004 [20,27,28], and the more recent dated from 2020 [23].

- Duration of the hypoxia programme

The duration of the normobaric hypoxia exposure programme and the sessions per week were variable (Table 3). Three of the studies had a programme duration of one week [21,26,28], while the maximum duration was thirteen weeks, which occurred in only one of the cases [24]. Regarding the sessions per week, half of the studies exposed the subjects to hypoxia every day [22,26,27,28,29,31], while the remainder ranged from two to five sessions per week.

- Participants

The sporting or professional levels to which the different populations of runners in the different articles belonged were highly heterogeneous (Table 2): 76 belonged to college teams, 18 were part of a local athletics team, 25 were national runners with the USA national team, 28 were considered elite, and there were 37 participants whose professional level was not specified. Mean VO_2_ max (ml/min/kg) was not included in every study, while the maximum was recorded as 68.75 and the minimum was 59.65. All the participants were middle- or long-distance runners. Only three of the studies described the timing of the participants undergoing the hypoxia programme: Katayama et al. [27] conducted their study seven weeks before the start of a championship, Hoshiwaka et al. [26] conducted theirs during the training season, while the study by Burstcher et al. [24] took place two months after the end of the season. 

The ages of the participants ranged from 19.6 to 33.4 years. In terms of gender, five of studies included men and women [20,21,24,30,31], six included only men [22,23,25,27,28,29] and in one study the participants were only women [26].

- Intervention

##### Intervention Model 

Normobaric hypoxia was administered to participants in two states: resting or performing physical activities (Table 3). 

Some of the studies included several intervention groups in which participants underwent normobaric hypoxia at rest and/or normobaric hypoxia while undertaking physical activity. The study by Robertson et al. [30] presented two intervention groups: the first was administered hypoxia during physical activity only, while the second was administered the treatment in both states, i.e., at rest and during physical activity. Similarly, Neya et al. [29] established two intervention groups: the first group underwent hypoxia at rest, and the second group underwent hypoxia during physical activity. The remaining studies administered hypoxia at rest only [20,22,23,24,27,28,31], during physical activity only [21,25] and both at rest and during physical activity [26]. 

Among the studies administering hypoxia at rest, four did not explain the specific physical position of participants at the time of administration [20,24,27,31]. In one group in the study by Neya et al. [29] and in the study by Brugniaux et al. [22], participants received hypoxia while sleeping. The hypoxia administrations lasted 10–12 h/session and 14 h/session, respectively. In two of the studies, participants were seated in a chair [23,28]. Regarding the form of exposure, Urymstev et al. [23] performed intermittent normobaric administration, with a duration of 10:10 per session, i.e., 10 min of normoxia followed by 10 min of hypoxia, while Katayama et al. [28] administered 3 h continuously. 

In studies by Robertson et al. [30] (second group) and Hoshiwaka et al. [26], participants were subjected to hypoxia in both states. The latter study administered normobaric hypoxia to subjects in the supine position for 7 h each night. In addition, it combined treadmill and cycloergometer exercises for physical activity. The second group in the study by Robertson et al. [30] also used the treadmill to exercise under hypoxic conditions for a duration of approximately one hour per session. However, for administering hypoxia at rest, the exact position used was not described.

Subjects in the Dufour et al. [25] and Hobbins [21] studies, and in the second intervention group of Neya et al. [29] and the first intervention group of Robertson et al. [30] underwent normobaric hypoxia while performing the physical activity, all using the treadmill to perform the activity. 

##### Physical Activity Programme during Exposure to Normobaric Hypoxia

The second group in the study by Neya et al. [29] trained on a treadmill for 30 min each session. The intensity was of 80–90% of the maximum heart rate (HRmax) reached during the VO_2_max test at sea level before the intervention. The subjects started at 80% of their HRmax for the first 10 min, and gradually increased speed over the next 20 min until they reached 90% of their HRmax.

The duration of the exercise in the physical activity programme of the Dufour et al. [25] study changed according to the week of intervention. During weeks 3 and 6, the participants ran for two periods of 12 min each, while during weeks 4 and 7, the duration of each running period was increased to 16 min, and during weeks 5 and 8, the participants ran for two periods of 20 min each.

Robertson et al. [30] presented two intervention groups. During the week they undertook one long-duration session, one moderate-duration session and two interval or high-intensity sessions. The intensity during each session is not described in two studies [25,30]. 

Hobbins et al. [21] suggested a HIIT (High Intensity Interval Training) programme during the hypoxia. This consisted of four running sessions of 4 min duration. Between each of the sessions there was a rest time of 3 min, also under normobaric hypoxia conditions. The intensity during the first 30 s of the HIIT was determined by the participant’s favourite running speed; after 30 s, the participant indicated an increase or decrease in the speed by signalling up or down with their thumb. Each participant was in normobaric hypoxia for 28 min per session. 

The training programme chosen by Hoshiwaka et al. [26] consisted of five sets of 30 s on a cycloergometer, 4 min rest between exercises, six periods of 5 min running at a heart rate of 120–180 beats per minute, and finally, 30 min of cycloergometer exercise at 80 rpm. 

##### Duration of Exposure to Hypoxia per Session and Duration of the Intervention 

The duration of hypoxia per session was variable among the different intervention groups, both at rest and during physical activity. Regarding the first group (at rest), two studies performed the exposure intermittently [20,23]. Julian et al. [20] conducted 5:5, i.e., 5 min of hypoxia followed by 5 min of normoxia for 70 min, 5 times per week for 4 weeks, while Urymtsev et al. [23] followed a pattern of 10:10 min in each session. The latter article provides no information about either the number of sessions per week or the total duration. 

Among the studies in which hypoxia was undergone while sleeping, Neya et al. [29] applied hypoxia for 10–12 h over 29 nights, Bryniaux et al. [22] followed a programme of 14 h per day for 18 days and Hoshiwaka et al. [26] administered hypoxia 7 h per day for 6 nights. In all cases, these sessions took place on consecutive nights. 

Regarding the studies [24,27,28,31] in which all participants were seated, or in which the position was not specified in the study, participants had an average of 5 h of exposure per session, and the hypoxia programme lasted an average of 29.4 days.

In relation to the groups that underwent hypoxia while they were performing physical activity, the mean duration of the sessions was 45 min and the hypoxia programme lasted a mean of 19.5 days. 

##### Altitude and Hypoxia Simulator Device

Hypoxia was generated using different altitude simulator devices. Face masks were used in 41.67% of studies [24,25,26,27,31] and hypoxic rooms were used in 58.63% [20,21,22,23,28,29,30]. 

The simulated altitudes varied widely between studies. In three studies [20,22,24], simulated altitudes changed during the intervention. Participants in the study by Julian et al. [20] were exposed to different altitudes throughout the 4 weeks of hypoxia. The first week the fraction of inspired oxygen was 0.12, in the second week it decreased to 0.11 and in the two last weeks it fell to 0.10. Brugniaux et al. [22] simulated an altitude of 2500 metres for the first six nights of the study, and 3000 metres for the following twelve nights. Similarly, Butscher et al. [24] varied the altitude by between 15% and 11%, which equated to fluctuations from 3200 to 5500 metres, respectively. Additionally, several authors chose to vary the altitude and oxygen percentage depending on the presence or absence of physical activity. Hoshiwaka et al. [26] administered an inspired oxygen fraction (FiO_2_) of 16.4% at rest and an FiO_2_ = 14.4% in the active state, i.e., equivalent to 2000 and 3000 metres, respectively. Robertson et al. [30] made a similar distinction, stipulating 3000 metres when performing physical activity and 2200 metres when at rest. Neya et al. [29], Dufour et al. [25] and Robertson et al. [31] stipulated 3000 metres for runners. Katayama et al. [27] and Katayama administrated an inspired oxygen fraction of 12.3%. Urymtsev et al. [23] did not specify the simulated altitude. 

### 4.2. Outcome Measure

Outcome measures were highly heterogeneous among the different included articles. This is because although all the studies administered normobaric hypoxia to middle- and long-distance runners, the objectives of some studies included highly specific outcomes. In this systematic review, we classified the outcome measures into two groups: haematological parameters (Table 4) and sport performance measures (Table 5).

#### 4.2.1. Sports Performance Measures 

These measures were executed by field trials or laboratory tests. We included time to exhaustion and 3000 m run tests in this group.

#### 4.2.2. Haematological Parameters

This section includes the following parameters: hemoglobin concentration, lactate concentration, percentage of reticulocytes, oxygen saturation, heart rate, maximal heart rate, percentage of hematocrit and erythropoietin values. 

## 5. Discussion 

As far as we know, this is the first systematic review evaluating the changes produced by exposure to normobaric hypoxia in middle- and long-distance runners from different competition levels. 

In terms of exposure time, most studies used exposure times of 3 h or less; this type of exposure is called intermittent hypoxia [16]. Currently, this appears to be the most commonly used type, as the articles with the oldest publication dates were those with the longest exposures (more than 12 h/day) [22,29,31].

The hypoxic room was the most commonly used hypoxia simulator. Studies with short exposure time (less than one hour) used a face mask [21,23,25]. This may be because a hypoxic room or tent is more comfortable for long exposure times.

Hoshiwaka et al. [26] was the only study in this review to use higher altitudes during exercise than at rest. This is striking, as exercise sessions in hypoxia place greater stress, fatigue, immunosuppression and stress on the body than those performed in normoxia [32], and coupled with the increased altitude, cause an even greater stimulus to the body.

### 5.1. Outcome Measures 

It seems that it is not necessary to apply long exposure times [29] to obtain a significant increase in the time to exhaustion, with interventions of three hours or less being effective [25,27], and the treatment’s effectiveness does not depend on the state of activity in which it is applied (at rest or exercise). The HIIT programme, carried out by Dufour et al. [25], increased this outcome measure in runners. In addition, Vallier et al. [33], reported that a HIIT programme under hypoxia conditions for 3 weeks also increased time to exhaustion in in elite triathletes. 

The study of Katayama et al. [27] was the only one to show a trend of improvement in the 3000 m run, while Julian et al. [20] showed no significant change. This may have been a consequence of using sessions that were too short in time (70 min), and in addition, they alternated between normoxic and hypoxia states, and thus did not achieve 70 min of hypoxia. Even so, some authors have achieved significant improvements in the 3000 m race time using hypobaric hypoxia [34]. However, we know that hypobaric hypoxia is a more stressful stimulus than normobaric hypoxia [35], so these parameters should be tested using normobaric hypoxia to ascertain whether there are similar improvements in 3000 m run times. 

To increase haemoglobin concentration, studies agreed on the need for long exposure times [36,37]. However, there was no consensus on the exact time, with some claiming that 12–16 h/day for 25 days are necessary [37], while others affirmed that 8–10 h/day are enough [36]. This may be the reason why the studies by Julian et al. [20], Katayama et al. [27] and Bustcher et al. [24] did not increase haemoglobin concentration (using short exposures), whereas the studies in our review using exposures of 14 h/day or more achieved an increase after intervention [30,31].

In the studies that showed an improvement in the reticulocyte percentage [20,30] or in haematocrit [24], improvement was only seen during the days following the start of the intervention; however, with the passage of time (during the intervention or after the intervention had ended) these improvements were not sustained. Julian et al. [20] increased the altitude week by week, and despite these variations in the stimulus, the increase in reticulocytes was not sustained after the end of the intervention. 

Oxygen saturation (SpO_2_) decreased in three studies [21,26,31]. Hoshiwaka et al. [26] intended to relate the effects of normobaric hypoxia to sleep parameters and, consequently, hypoxia occurred while the patients were sleeping. This study evinced that exposure to hypoxia causes a decrease in the SpO_2,_ and this in turn leads to lighter sleep. This finding adds to others in the literature [38]. This may suggest that the application of a hypoxia programme while athletes sleep may affect the quality of their rest. However, we cannot relate this to a reduction in their sports performance, because in their study, Hoshiwaka et al. [26] did not take into account field or laboratory tests evaluating the participants’ sports performance. 

The decrease in SpO_2_ in the study by Hoshiwaka et al. [26] was related to an increase in heart rate during the first night after breathing oxygen-poor air compared to normoxia. However, when comparing the last night of hypoxia with the first night, there was a significant decrease, which derived from the effects of training on the autonomic nervous system, and not from the acclimatisation to hypoxia. Acclimatisation of the autonomic nervous system took place in six days in the study by Hoshiwaka et al. [26] (from the first to the sixth night of hypoxia), and the same was true for the study by Astorino et al. [39]. 

Of the articles included in this review, EPO values only increased in the study by Robertson et al. [31], whose duration of hypoxia administration was 14 h/day, and whose participants were elite middle- and long-distance runners. It increased on days 2 and 6 of exposure (in both block 1 and 2) but was not maintained on days 20 and 27 in either intervention block. In the studies by Katayama et al. [27] and Julian et al. [20], 3 h/day and 70 min/session, respectively, were not enough to stimulate a sharp increase in the concentration of EPO. These results in the studies in this review are ratified by the literature, as many authors defend the idea that the use of daily exposure sessions shorter than 12 h does not produce any improvement in the production of red blood cells in athletes. [36,40,41,42].

### 5.2. Limitations, Perspective and Practical Applications

Limitations of this review include the fact that there was no control group in any of the studies. Another limitation is that not all studies in this systematic review included male and female participants. Six studies only included male runners. This may have an impact on the results, as the effects after hypoxia may be different depending on the sex of the participant.

Only three studies took into account the time of the season when the hypoxia exposure took place. This is an important factor to consider in future research, as depending on the time of the season, participants may have a greater or lesser range of improvement, which may influence their athletic performance. 

In addition, future studies may consider the relationship between exposure to hypoxia during sleep and sports performance. Sleep parameters have been shown to be significantly altered, but it has not been demonstrated whether this has any impact on athletic performance.

The strengths of this review are that it is focused on runners only, excluding other endurance athletes, such as cyclists, thus differentiating it from other studies. In addition, it includes a large number of haematological parameters and performance measures. This synthesis of information may help coaches who use hypoxia in their preparation.

## 6. Conclusions

Short exposures (less than 3 h) to normobaric hypoxia significantly increase the time to exhaustion. The methodological quality of the studies that report this is 5/10 and 6/10 on the PEDro scale.

This systematic review shows that studies using long periods of exposure to hypoxia (14 h or more) increased haemoglobin values, while short exposure times were ineffective. This assertion was made by several studies in this review, including the study with the highest score on the PEDro Scale (7/10).

Altitudes and durations of exposure to hypoxia were highly heterogeneous in the included studies. These values will differ according to the haematological parameters to be improved. However, the most commonly used altitude is 3000 m. 

## Figures and Tables

**Table 1:** Methodological quality (PEDro scale).

Name, Year	Scores for Each of the Items											Score
	EC	RA	CA	BC	BS	BT	BA	AF	ITTA	BGC	PEAV	
Brugniaux et al. [22]	No	No	No	No	No	No	No	Yes	Yes	Yes	No	3/10
Burtscher et al. [24]	No	Yes	No	Yes	No	No	No	Yes	Yes	Yes	No	5/10
Julian et al. [20]	No	No	Yes	Yes	Yes	Yes	Yes	Yes	Yes	No	No	7/10
Dufour et al. [25]	No	Yes	No	Yes	No	No	No	Yes	Yes	Yes	Yes	6/10
Hobbins et al. [21]	No	Yes	No	Yes	Yes	No	No	Yes	Yes	Yes	Yes	7/10
Hoshiwaka et al. [26]	No	No	No	No	No	No	No	Yes	Yes	Yes	Yes	4/10
Katayama et al. [27]	No	Yes	No	Yes	No	No	No	Yes	Yes	Yes	No	5/10
Katayama et al. [28]	No	No	No	Yes	No	No	No	Yes	Yes	Yes	No	4/10
Neya et al. [29]	No	Yes	No	Yes	No	No	No	Yes	Yes	Yes	No	5/10
Robertson et al. [30]	No	Yes	No	Yes	No	No	No	Yes	Yes	Yes	No	5/10
Robertson et al. [31]	No	No	No	No	No	No	No	Yes	Yes	Yes	Yes	4/10
Uryumtsev et al. [23]	No	No	No	No	No	No	No	Yes	Yes	Yes	No	3/10

EC: Eligibility criteria; RA: Random allocation; CA: Concealed allocation; BC: Baseline comparability; BS: Blind subjects; BT: Blind therapists; BA: Blind assessors; AF: Adequate follow-up; ITTA: Intention-to-treat analysis; BGC: Between-group comparisons; PEAV: Point estimates and variability.

**Table 2:** Study characteristics.

Name, Year	*n*	Sex	Age	VO_2_ Max mL/kg/min	Duration	Session [Day/Week]	Intervention Moment	Sport	Sport Level
Brugniaux et al., 2006 [22]	11	M	23.5	63.3	18 days	7 days/week	-	Middle-distance runners	Elite
Butscher et al., 2010 [24]	11	M F	21.8	59.65	13 weeks	3 days/week	2 months after the end of the season	Middle-distance runners	National level
Julian et al., 2004 [20]	14	F M	25	4.9625 mL/min	4 weeks	5 days/week	-	Middle-and-long-distance runners	National level
Dufour et al., 2006 [25]	18	M	30.3	62.85	6 weeks	2 days/week	-	Long-distance runners	Local athletic teams
Hobbins et al., 2019 [21]	19	M F	33.4	-	1 week	3 days/week	-	-	-
Hoshiwaka et al., 2013 [26]	7	F	19.6	-	1 week	7 days/week	During the season	Middle-distance runners	Intercollege level
Katayama et al., 2004 [27]	15	M	22.2	-	2 weeks	7 days/week	7 weeks before the championship.	Long-distance runners	Intercollege level
Katayama et al., 2004 [28]	29	M	21.05	-	CONT_1_:1 week INT_2_: 2 weeks CONT_2_:2 weeks	7 days/week	-	Endurance runners	Intercollege level
Neya et al., 2007 [29]	25	M	20.6	60.3	31 days	INT_1_: 7 days/week INT_2_:7 days/week	-	Middle-and-long-distance runners	Intercollege level
Robertson et al., 2010 [30]	17	M F	-	65.5	3 weeks	4 days/week		Middle-distance runners	-
Robertson et al., 2010 [31]	16	M F	-	68.75	6 weeks	7 days/week		Middle-and-long-distance runners	Elite
Uryumtsev et al., 2020 [23]	20	M	21.5	-	-	-	-	Middle-distance runners	-

INT1: Group 1 of intervention; INT2: Group 2 of intervention; CONT1: control group 1; CONT2: control group 2; F: female; M: male.

**Table 3:** Characteristics of normobaric hypoxia.

Name, Year	Exposure Type	Exposure Time	Altitude Simulated/Saturation	Administration Hypoxia	Hypoxia Moment	Other
Brugniaux et al., 2006 [22]	INT_1_: HR (sleeping) CONT_1_: no hypoxia	INT_1_: 14 h/day	HR: (FiO_2_ = 6 nights at 2500 m/0.174, 12 nights at 3000 m/0.164). CONT_1_:1200 m	Hypoxic room	-	INT_1_ and CONT trained at 1200 m normoxia
Butscher et al., 2010 [24]	INT_1_: HR (position not described) CONT_1_: no hypoxia	2 h session/3 times per week for 10 weeks	FiO_2_ = 15%(3200 m)–11% (5500 m)	Hypoxic room	-	-
Julian et al., 2004 [20]	INT_1_: HR CONT_1_: no hypoxia	5:5 min during 70 min, 5 times a week.	INT_1_: FiO_2_ changed from week 1 to week 4, was: 0.12, 0.11, 0.10, 0.10, respectively. CONT_1_: FiO_2_ = 0.209	Hypoxic room	1–2 h after or before exercise training	-
Dufour et al., 2006 [25]	INT_1:_ HE. Different each week; 24 to 40 min in treadmill CONT: HE	Week 3 and 6: 24 min/session Week 4 and 7: 32 min/session Week 5 and 8: 40 min/session	INT_1:_ (FiO_2_ = 14.5%) o 3000 m CONT_1_: (FiO_2_ = 20.9%)	Facemask	-	-
Hobbins et al., 2019 [21]	INT_1_: HE (4 × 4 min running × 3 min at rest (28 min total hypoxia) CONT_1_: no hypoxia	28 min/session. (2 sesions)	INT_1_: (FiO_2_ = 15% o 2700 m) CONT_1_:(FiO_2_ = 20.9%)	Facemask	-	-
Hoshiwaka et al., 2013 [26]	INT_1_: HR (sleeping) and HE (cyclo-ergometer and treadmill)	HR: 7 h/night (6 nights) HE: 1 h aprox/session	HR: (FiO_2_ = 16.4% or 2000 m) HE: (FiO_2_ = 14.4% or 3000 m)	HR: hypoxic room HE: (not indicated)	-	-
Katayama et al., 2004 [27]	INT_1_: HR CONT_1_: no hypoxia	3 h/session during 14 consecutive days.	INT_1_: (FiO_2_ = 12.3%) CONT_1_: no hypoxia	Hypoxic tent	-	
Katayama et al., 2004 [28]	INT_1_ and CONT_1_:HR (sitting) INT_2_ and CONT_2_: HR (sitting)	INT_1_ and CONT_1_: 3 h/day during 1 week INT_2_ and CONT_2_: 3 h/day during 2 weeks	INT_1_ and INT_2_: FiO_2_= 12.3–12% CONT_1_ and CONT_2_= normal FiO_2_ (no hypoxia)	Hypoxic tent	-	-
Neya et al., 2007 [29]	INT_1_: HR (sleeping) INT_2_: HE 30 min treadmill CONT_1_: HE	INT_1_: 10–12 h during 29 nights. INT_2_: 30 min during 12 days CONT_1_: no hypoxia	INT_1_:3000 m (FiO_2_ = 0.144) INT_2_:3000 m (FiO_2_ = 0.144)	Hypoxic room INT_1_: 50 m^3^ INT_2_: 100 m^3^	-	-
Robertson et al., 2010 [30]	INT_1_: HE (treadmill) INT_2_: HR y HE (treadmill)	INT1: 4–5 h hypoxia in exercise/week INT2: 4–5 h hypoxia in exercise/week +14 h per day 3000 m rest	HE: 2200 m HR: 3000 m	Hypoxic room	-	-
Robertson et al., 2009 [31]	INT_1_: HR (Not described) CONT_1:_ no hypoxia	INT_1_:14 h/day	INT_1_:(FiO_2_ = 3000 m) CONT_1_:resided near sea level (600 m)	Hypoxic room	-	
Uryumtsev et al., 2020 [23]	INT_1_: HR (sitting)	10:10 min	(FiO_2_ = 10%)	Facemask	-	-

INT1: Group 1 of intervention; INT2: Group 2 of intervention; CONT: Control group; HR: Hypoxia at rest; HE: Hypoxia during exercise.

**Table 4:** Haematological parameters outcome.

Name, Year	Maximal Heart Rate or Heart Rate	Hemoglobin Concentration	Percentage of Hematocrit	Lactate Concentration	Percentage of Reticulocytes	Erythropoietin Values	Oxygen Saturation
Brugniaux et al., 2006 [22]	HRmax: No significant difference between the groups.						
Butscher et al., 2010 [24]		Significantly increased during the 5th week in comparison to the pre-intervention data, but this improvement did not remain during weeks 8 and 13. There were no significant differences between the groups (*p* > 0.05)	Significant increase in the intervention group during the 5 weeks of training in comparison with the values obtained before starting the intervention. However, there were no significant changes when comparing the two following measures during weeks 8 and 13 with the values from pre-intervention (*p* > 0.05).				
Julian et al., 2004 [20]	No significant difference between the groups	No significant differences between the values obtained after the intervention and those obtained pre-intervention (*p* > 0.05)		No significant changes (*p* > 0.05)	Significant increase in both groups after 12 days of intervention in comparison to 10 days after finishing (*p* < 0.05)	The group exposed to hypoxia obtained a significant decrease in comparison to the value before starting (*p* < 0.05)	
Dufour et al., 2006 [25]	HR max: no significant difference between the groups			No significant changes (*p* > 0.05)			
Hobbins et al., 2019 [21]				Major increase in INT_1_ (*p* < 0.05).			Significantly smaller (*p* < 0.01) in INT_1_ group in comparison to the CONT_1_ group.
Hoshiwaka et al., 2013 [26]	HR: values on the 1st night under hypoxia compared with those under normoxia indicated a significant increase after hypoxia (*p* < 0.05).						Decreased on the first night sleeping in oxygen-poor air conditions and on the sixth night (the last one of intervention), in comparison to the night in normoxia (*p* < 0.05).
Katayama et al., 2004 [27]	HR max: no differences between the groups (*p* > 0.05) HR: decreased (*p* < 0.05) after IH. Decreased (first to final night under hypoxia) (*p* < 0.05)	Any significant rise was produced in that measure after the intervention. No significant differences between groups (*p* > 0.05)	No significant difference in this outcome measure for the INT_1_ group after the hypoxia, and no significant differences between the groups (*p* < 0.05)		No significant improvements in favour of INT_1_ group, and no significant improvements found when comparing this group with the CONT_1_ group (*p* < 0.05).	No significant changes in the INT_1_ group after the exposure and no significant changes between the groups (*p* < 0.05)	
Katayama et al., 2004 [28]							INT_2_ values increased after the end of the intervention (*p* < 0.05). However, after 2 weeks the values were the same as the initial values. INT_1_ increased significantly after the end of the intervention (*p* < 0.05)
Neya et al., 2007 [29]	HR: no significant differences in the INT_1_ and INT_2_ after being exposed to hypoxia (*p* < 0.05)			No significant changes (*p* > 0.05)			
Robertson et al., 2010 [30]		Compared with INT_1_, INT_2_ had substantially higher values at week 1, 2 and 3.		No significant changes (*p* > 0.05)	INT_2_ substantially increased during weeks 1 and 3. No data for week 2. INT_1_ had no substantial changes in percent reticulocytes (*p* > 0.05).	INT_1_ had no substantial changes in percent reticulocytes.	
Robertson et al., 2010 [31]		INT_1_ had substantially higher values after block 2 than CONT_1_. After block 1 the differences were negligible.				Significant increase in INT_1_ during days 2 and 6 of exposure, both in block 1 and 2. Even so, these effects did not remain during days 20 and 27 in either of the two blocks of the intervention.	
Uryumtsev et al., 2020 [23]	HR: NT_1_ increased significantly (*p* < 0.05) during hypoxia by 31%.						INT_1_ decreased (*p* < 0.05) during hypoxia by 21%

INT1: Group 1 of intervention; INT2: Group 2 of intervention; CONT1: Control group; EPO: erythropoietin; HR: heart; IH: intermittent hypoxia; HRmax: maximal heart rate; HR: heart rate.

**Table 5:** Sport performance outcome.

Name, Year	3000 m-Run:	Time to Exhaustion
Julian et al., 2004 [20]	Did not improve after hypoxia exposure (*p* > 0.05)	
Dufour et al., 2006 [25]		Significantly improved (*p* < 0.05)
Katayama et al., 2004 [27]	Improving trend after intervention (*p* = 0.06)	Significantly improved (*p* < 0.05)
Katayama et al., 2004 [28]		
Neya et al., 2007 [29]		The groups exposed to hypoxia trend to significance (*p* = 0.07)

## Data Availability

Not applicable.
